# Associations among Primary Stability, Histomorphometric Findings, and Bone Density: A Prospective Randomized Study after Alveolar Ridge Preservation with a Collagen Cone

**DOI:** 10.3390/dj8040112

**Published:** 2020-10-02

**Authors:** Sigmar Schnutenhaus, Werner Götz, Jens Dreyhaupt, Heike Rudolph, Ralph G. Luthardt, Cornelia Edelmann

**Affiliations:** 1 Center of Dentistry, Dr. Schnutenhaus MVZ GmbH, 78247 Hilzingen, Germany; 2 Center of Dentistry, Department of Prosthetic Dentistry, Ulm University, 89081 Ulm, Germany; heike.rudolph@uniklinik-ulm.de (H.R.); ralph.luthardt@uniklinik-ulm.de (R.G.L.); edelmann@schnutenhaus.de (C.E.); 3 Oral Biology Laboratory, Department of Orthodontics, University of Bonn, 53113 Bonn, Germany; w.goetz@uni-bonn.de; 4 Institute of Epidemiology and Medical Biometry, Ulm University, 89081 Ulm, Germany; jens.dreyhaupt@uni-ulm.de

**Keywords:** alveolar ridge preservation, dental implant, primary stability, DRKS00004769

## Abstract

Background: The objective of this investigation was to examine whether determination of bone density (BD) with a cone beam computed tomography (CBCT) scan could help predict the primary stability (PS) of the implants and to investigate whether associations between the histomorphometric findings and the CBCT scan could be observed. Materials and methods: In this randomized clinical study, the efficacy of alveolar ridge preservation (ARP) with a combination of a collagen cone and a collagen membrane procedure after tooth extraction was investigated. CBCT scans were obtained after a healing period of 8 (±1) weeks. Subsequently, the CBCT scans were evaluated in terms of BD at different heights of the former socket. Eleven (±1) weeks after tooth extraction, implant placement was performed and PS was measured with resonance frequency analysis. Potential associations among the radiologically measured BD, the histomorphometric results, and the PS were analyzed. Results: No direct association was observed between the radiologically determined BD and the histomorphometric findings. No significant associations could be found between the BD and the PS. Conclusion: No significant associations were observed among the BD determined by the CBCT, the histomorphometric findings, and the PS.

## 1. Introduction

Primary stability of implants is considered a prognostic factor for osseointegration. Concepts of early implant loading also require sufficiently high primary stability. Therefore, information about bone quality obtained beforehand using measures such as cone beam computed tomography (CBCT) could be helpful.

For the evaluation of bone density in Hounsfield units (HUs), computed tomography (CT) scans have to be obtained, as CBCT scans with reduced radiation exposure are not suitable [1]. CBCT scans have limitations while analyzing the quantitative data of bone density measurements [2]. Furthermore, the measurement of bone density depends significantly on the CBCT device, and on the scanning device settings [3]. Neighboring structures such as metallic prosthetic restorations influence the bone density measured by a CBCT scan [4]. Since the bone density values from a CBCT scan are associated with those from a CT scan [5], the expected bone quality can be estimated using CBCT values before the implant procedure [6]. Thus, a pre-implant evaluation of bone quality can be performed. The measured HUs are conveyed to the CBCT scan [7]. Algorithms for record calculation have been developed over the past few years. The grey values of a CBCT scan can be converted to mineral density by a calibration curve specific to the CBCT device [8]. To date, scarce clinical data are available regarding the association between the bone quality measured by CBCT and the histomorphometric findings. Studies involving animal experiments [9] or human carcasses [10] have provided the initial findings.

The alveolar process undergoes resorptive changes after tooth extraction. In addition to the increased surgical effort in terms of necessary grafting measures and higher treatment costs, the loss of bone volume can also compromise functional and esthetic results [11]. Different actions can be performed after tooth extraction to influence bone and soft tissue healing and, consequently, to prevent bone loss. Various clinical concepts are being proposed, including those other than the insertion of different bone substitutes. These methods of alveolar ridge preservation (ARP) showed a significant reduction in bone loss [12]. Though the implants placed in the regenerated parts of the alveolar ridge show similar survival rates to the implants placed in native bone, it was not possible to determine whether a particular augmentation technique was superior to others based on implant survival rates [13]. Moreover, no clear evidence was observed regarding the superiority of a particular ARP measure in terms of new bone formation [14] or preservation of bone and keratinized tissue dimensions [14].

Since ARP measures change not only the quantity of bone but potentially the quality of bone [15], further investigations are needed. However, it is not currently possible to make statements on clinical relevance. The duration of time between the ARP measure and the implant placement must be considered an important factor. The change in bone density before and after the ARP measures has been calculated in addition to metric analysis of bone levels [16]. The analysis of CBCT scans regarding radiologically measured bone density was introduced as a method to evaluate the ARP measures [17].

The primary stability of an implant is considered an indication of bone quality. Various authors have found an association between high primary stability and secondary stability [18]. These findings have led to the suggestion that the primary stability of an implant is a key parameter while evaluating the implant insertion prognostically [19]. Primary stability also serves as a diagnostic parameter for ARP measures [20]. In addition to bone quality, primary stability is also dependent on surgical techniques and, especially, on the implant design [21]. Numerous studies have shown that bone density measured with a CBCT scan was associated with primary stability of implants measured with resonance frequency analysis (RFA) [22,23].

In a recent clinical and histological study, we demonstrated that there were no significant differences in terms of new bone formation and bone quality when use of a combination of a collagen cone and a collagen membrane after tooth extraction was compared with a control group without ARP. However, different descriptive findings such as increased bone remodeling, increased osteoblast activity, and increased vascularization were observed [15]. Furthermore, the storage of the collagen had no negative effects on the histological level. On the other hand, significant preservation of the alveolar ridge was demonstrated in the area of the maxillary anterior teeth [24].

The primary aim of the present study was to determine the possible associations among bone density measured by CBCT, the histomorphometric observations, and the primary stability of implants.

Furthermore, the influence of a combination of a collagen cone and a collagen membrane on the healing process of extraction sockets was examined in terms of bone density and primary stability.

## 2. Materials and Methods

The study design corresponded to the design described in the previously published article by Schnutenhaus et al. [24]. The present trial was performed as q parallel-group randomized human clinical trial in accordance with the Declaration of Helsinki. After approval from the Ethical Committee of Ulm University (No. 337/12, 13 February 2013) the study was registered in the German Clinical Trials Register as DRKS00004769 (International Clinical Trials Registry Platform of the World Health Organization).

The study participants were informed about the study before their participation orally as well as in writing and a written informed consent was obtained from all participants.

### 2.1. Participants

**Figure 1:**
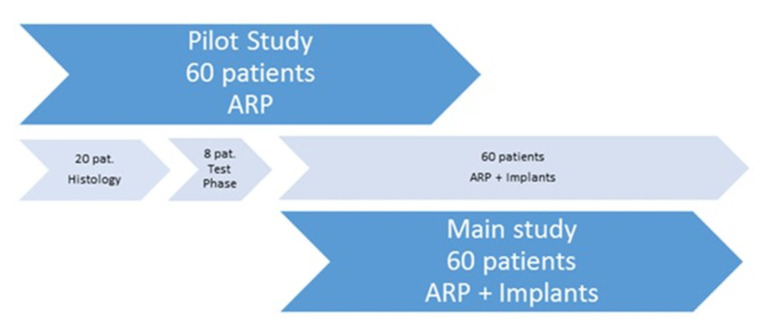
Division of patients into the pilot study and the main study. ARP: alveolar ridge preservation.

In total, 88 patients with at least one tooth in the upper jaw indicated for extraction and replacement by an implant-supported prosthetic restoration participated in the study. Patients were consecutively divided into two groups (the test group and the control group, 44 patients each). Participants in the pilot study and the main study were divided into three subgroups. Twenty patients underwent histological examination [15]. Eight patients were included in the test of an implant insertion tool. Sixty patients were included in the main study for implant placement with a modified implant drill system and follow-up examination (Figure 1). The division into subgroups was necessary because the drilling protocol of the manufacturer was violated by sampling for histological examinations. Furthermore, two different versions of implants were used (Camlog Guide 1.0 vs. 2.0).

Forty-four patients received a combination of a collagen cone and a collagen membrane (Parasorb Sombrero^®^; Resorba, Nürnberg, Germany), which was inserted into the alveoli after tooth extraction (test group). For patients in the control group, wound healing was allowed to proceed without further intervention. Inclusion and exclusion criteria are listed in detail in the previously published article by Schnutenhaus et al. [24]. The clinical therapy steps were carried out between February 2013 and March 2015.

### 2.2. Treatment Protocol

The monocentric study was conducted at the private practice of the first author (SIS), who exclusively performed all interventions and follow-ups. All participating patients were recruited from the same private practice in Hilzingen, Germany.

Interventions on the day of tooth extraction included local anesthesia with articaine (Ultracain DS 1:200,000; Sanofi Aventis, Frankfurt, Germany), atraumatic extraction of the teeth after complete mobilization, curettage of the extraction socket, and no further measures for the patients in the control group. Patients in the test group received a combination of a collagen cone and a collagen membrane according to the manufacturer’s instructions. All patients were given post-extraction instructions for the 24 h after tooth removal. One week later, visual inspection of all wounds was performed.

A nonsteroidal anti-inflammatory drug (600 mg ibuprofen) was prescribed for pain reduction, to be taken as needed. No systemic prophylactic antibiotic was performed.

After 11 (±1) weeks post extraction, implants were placed (Conelog Guide 1.0 (8 patients, Conelog 2.0 (80 patients), Camlog Biotechniologies, Basel, Switzerland). Implant positions were determined by using implant planning software (SMOP; Swissmeda, Zürich, Switzerland). The positioning of the implants was driven by restorative criteria. The treatment plan was implemented in a template-driven approach using a 3D-pronted drilling stent.

### 2.3. Radiological Measurement of Bone Density

Overall, 88 patients were enrolled in the study. CBCT scans were used to analyze bone density in the region of the extraction socket after a healing period of 8 (±1) weeks (T1).

**Figure 2:**
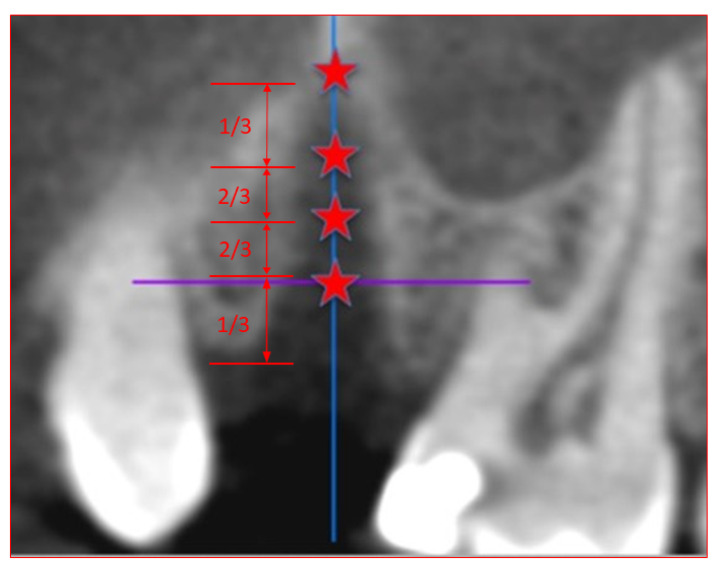
Definition of the measuring points in the cone beam computed tomography scan. The measuring points were located at the junction of coronal third and lower two thirds in the middle and at the junction of apical third and the upper two thirds of the alveoli. Measuring points were arranged on an axis in the center of the alveoli.

**Figure 3:**
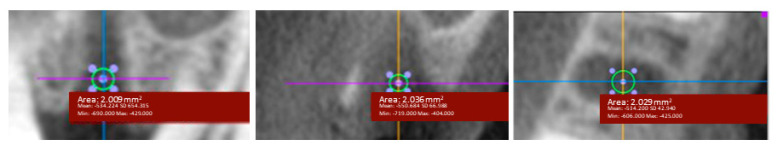
Determination of the region of interest (ROI) with an area of 2 mm2 in three different planes (axial, sagittal, and coronal). Circular areas of 2 mm2 designated as ROIs were marked.

CBCT scans for the evaluation of bone density were loaded into the software OsiriX Lite Version 9.0 (Pixmeo SARL, Bernex, Switzerland). The dentists analyzing the scans were calibrated prior to the evaluation. Each measurement was performed with the same computer at a single workspace. Measurements were performed at three different heights of the extraction sockets. The first measurement was at the junction of the coronal third and the lower two thirds, the second was in the middle, and the third measurement was at the junction of the apical third and the upper two thirds of the alveoli. A fourth measurement of the local bone was further made apical and palatal to the alveoli (Figure 2). For each measurement, a region of interest with an area of 2 mm^2^ was placed in the center of the alveoli (Figure 3).

After data collection using Microsoft Excel 2013 (Microsoft Corporation, Redmond, WA, USA), all statistical analyses were performed with SAS^®^ Version 9.4 (SAS Institute, Cary, NC, USA). Average values of bone density were collected from the three image planes at each level of the alveoli. Values were adjusted for further analysis. The measured values of the palatal-apical dimensions of the local bone were used as reference values.

### 2.4. Primary Stability of the Implants

All implants were inserted using a template-guided procedure according to the study protocol and the manufacturer’s instructions. No further modification in the implant position was made, as the accuracy of this procedure was analyzed in a subsequent study. The primary stability was measured immediately after the removal of the surgical template. Insertion posts were removed carefully while stabilizing the implant with a counter ratchet to prevent derotation. The implant stability quotient (ISQ) values were measured with Osstell^TM^ (Integration Diagnostics AB, Göteborg, Sweden), a device that uses RFA technology. Two measurements from two different directions (mesial and buccal) were collected for each implant.

### 2.5. Association between the Primary Implant Stability and the Radiological Bone Density Measurement

A positive association between the primary implant stability and the radiologically measured bone density was expected. The results of the association analysis were expected to serve as a reference for the expected primary stability after CBCT analysis.

### 2.6. Histomorphometric Evaluation

A semiquantitative evaluation was performed based on the qualitative characteristics. The first step was to test whether the features were recognizable. The results were then classified semi-quantitatively. As a result, a yes/no decision was logged.

The samples were evaluated for osteogenesis, remodeling, osteoblast activity, discernible calcification/mineralization, detection of bundle bone in the alveoli, vascularization of the alveoli, and inflammation. Materials and methods are described in detail in the previously published article by Schnutenhaus et al. [15]. The number of cases with 10 test and 10 control samples were based on the specifications of the ISO10993-6 [25].

### 2.7. Randomization

A randomization list was created for the entire study (Institute of Epidemiology and Medical Biometry, University of Ulm, Ulm, Germany). Assignment to the various groups was made in six layers. The data were stratified as follows:

- According to sex (2 groups: male and female)
- According to the region of the test tooth (3 groups: anterior, premolar, and molar)

A responsible person informed the study center in which group of patients was classified according to the randomization list.

### 2.8. Blinding

Blinding of the socket treatment was not possible due to the study design. However, CBCT images taken at T1 were forwarded to the analyst (CE) in blinded and anonymized form. The histological evaluation (WG) was also blinded. Unblinding was performed only after the completion of the analysis, documentation, and statistical analysis.

### 2.9. Statistical Analysis

For the continuous variables, minimum, median, and maximum values were reported. Nominal and ordinal features were described with their absolute and relative frequencies.

Differences between the test and the control groups were investigated using the Wilcoxon rank-sum test. Due to the exploratory nature of this study, all statistical results were hypothetical in nature and should not be interpreted as confirmatory. All statistical tests were carried out at level α = 0.05 (two-sided). No adjustments were made for multiple testing.

The association analysis of the measured ISQ values and the adjusted values of bone density measurements was performed using scatter plots and Spearman’s rank correlation coefficient for two continuous variables and the point-biserial correlation coefficient for continuous and binary variables.

Due to a lack of clinical data, sample size could not be estimated in advance. Therefore, this study as performed as an explorative study.

## 3. Results

### 3.1. Study Population

This study included 88 patients for the collective analysis. One drop-out was a patient who did not show up for tooth extraction after having been recruited. All patients were treated according to the clinical protocol without any post-operative complications. All included patients completed the trial. Patients were assigned to groups in six strata. Forty-four patients were randomized to be treated with the collagen material and 43 patients served as controls, receiving no further interventions after tooth extraction.

Fifty-two female and 35 male patients participated in this study. The ARP group consisted of 27 female and 17 male patients, while the control group consisted of 25 female and 18 male patients. The mean patient age was 49.6 years (range: 21.7–77.3 years). In the histomorphometric study 10 female patients and 10 male patients were included. The mean age of this patients was 46.6 (21.9–71.4) years. Randomized distribution of the teeth according to their position in the jaw is shown in Table 1.

### 3.2. Changes in the Radiologically Measured Bone Density after ARP

Table 2 shows the parameters of the radiological bone density as a function of localization of the measuring points in the alveoli for the ARP and the control groups. The mean values were reported for each sectional plane. The values were standardized with the respective value of alveolar bone density measured in the palatal-apical direction. This procedure allowed generation of “Hounsfield-equivalents.” The Wilcoxon rank-sum test showed no significant differences between the ARP group and the control group for any of the regions.

In contrast, significant differences were found among the bone densities at different sectional planes (Table 3). The bone density increased from the coronal measuring points to the more apically located measuring points. The difference of 0 analyzed by Wilcoxon signed-rank test was *p* < 0.01 for all comparisons. There were no significant differences between the ARP group and the control group.

### 3.3. Association between Radiologically Measured Bone Density and Histomorphometric Results

For the analysis of potential associations between the radiologically measured bone density and the histomorphometric results, scatter plots were applied and Spearman’s rank correlation coefficient was calculated for histomorphometric parameters with more than two manifestations. When two manifestations or yes/no records were present, the point-biserial correlation coefficient was calculated (Table 4). No parameter showed significant differences (*p* < 0.05). Descriptive data revealed pronounced remodeling and higher bone density at the more apically located measuring points. Stronger vascularization or signs of inflammation were associated with reduced bone density at all the sectional levels.

### 3.4. Primary Stability of Implants

Sixty patients from the main study group were included for the evaluation of the primary stability. There were five dropouts including one patient who missed his appointment for tooth extraction after having been recruited (control group), three patients with implant placement failure due to clinically insufficient primary stability (control group), and one patient who had to cancel the treatment due to severe illness (ARP group). All other patients were treated according to the clinical protocol. No postoperative complications were observed.

After stratified randomization according to sex and the region of the studied tooth, 29 patients received ARP measures after tooth extraction. No interventions were performed after tooth extraction in the control group consisting of 26 patients.

Thirty-four female and 21 male patients participated in this study. The ARP group consisted of 19 female and 10 male patients, while the control group consisted of 15 female and 11 male patients. The mean patient age was 51.5 years (range: 24.0–77.0 years). Randomized distribution of the teeth according to their positions in the jaw is shown in Table 5.

The distribution of implant lengths and the diameters for both groups is presented in Table 6.

### 3.5. Changes in the Primary Stability after ARP

Table 7 demonstrates the descriptive data for the primary stability. Primary stability was measured in the buccal and in the mesial direction. No significant differences were found in the mean values between the ARP group and the control group.

### 3.6. Association between the Primary Stability and the Radiologically Measured Bone Density

We also examined whether there was an association between bone density calculated from the CBCT scan and the primary stability of the implants. Table 8 shows the association analysis of these two parameters. Spearman’s rank correlation coefficient was calculated.

No significant correlations were observed between radiologically measured bone density and the primary stability of the implants. Descriptive data showed higher bone density values and greater primary stability at the more apically located measuring points.

## 4. Discussion

### 4.1. Measurements of Bone Density

No significant difference was observed in the radiologically measured bone density using CBCT scan between the ARP group and the control group. The method applied in the present study is suitable for recording the healing process of the alveolus. As expected, bone density increased significantly from coronal end to apical end. However, no significant difference was found in bone density between the ARP group and the control group at any measurement point of the former socket.

In a previous clinical study, the effect of two ARP measures (a demineralized bovine material and a self-expanding composite) was compared after six months. Results showed significant differences in the radiologically measured bone density in the apical and the middle parts of the alveolus [26]. Implant placement three months after the extraction represents a clinically proven concept [27]. This period was also selected in the present study. During this period, new bone formation was found histologically [15]. However, mineralization that has an impact on the radiologically assessable bone density had not yet occurred. Radiologically measured differences in the density can also be influenced by residual bone grafting material. In the present study, this error was avoided, as the ARP material was completely absorbable. Histological investigations on bovine material have demonstrated that 30.8% of the overall bone volume consisted of the bone substitute nine months after the ARP measure [28].

Determination of bone quality with the help of CBCT scans shows reasonable reliability and validity. Further histomorphological evaluations to analyze the trabecular structures or to calculate the bone volume in proportion to the overall volume can be achieved using micro CT scans [29]. In an animal study, no association could be observed between bone density measured using a CBCT scan and the bone quality in the upper jaw as opposed to the lower jaw [30]. This fact might be another possible limitation of this procedure. Pauwels et al. also suggested that the attempt to correct the gray values of a CBCT scan is not sufficient for bone quality assessment [1].

Effects of ARP measures on bone density can be evaluated with CBCT scans obtained for implant planning if values are normalized by means of a control group [31]. Salimov et al. identified a significant association between bone density measured using a CBCT scan and the primary stability of implants measured using RFA. They suggested that CBCT scans are suitable to predict the implant stability and, therefore, help decide whether immediate or early loading of implants should be performed [22]. CT scans do not appear to have any advantage over CBCT scans in classifying bone quality [32]. The mean gray values obtained by a CBCT scan and HUs in a CT scan show a strong correlation [33]. Contrary to results of the present investigation, the study by Batsami et al. showed that the gray values from a CBCT scan of rabbits can be used to assess bone quality and the amount of new bone formation before implant placement [34]. Gonzáles-Garcia and Monje found a significant correlation between the gray values of a CBCT scan and the bone density in biopsies of the upper jaw. Therefore, they believed that clinical bone quality can be assessed using a pre-operative CBCT scan [35].

### 4.2. Primary Stability of Implants

In the present study, the insertion of a collagen cone with a collagen membrane did not significantly improve the primary stability of implants placed 11 weeks after the extraction. Results of RFA in the present study are similar to previously published values. Lozano-Carrascal et al. found average ISQ values of 67.2 ± 4.42 and 49.2 ± 15.3 for tapered and cylindrical implants, respectively [36]. The macro-design of the implants used in the present investigation was conical. Implants with diameters of 3.3 mm, 3.8 mm, or 4.3 mm and with lengths of 7 mm, 9 mm, 11 mm, and 13 mm were placed. According to Ohta et al., the diameter of conical implants does not influence implant stability [37], whereas the implant length is positively associated with the ISQ values of conical implants [38].

Winter et al. performed a finite element analysis of different factors influencing primary stability. The analysis included implant length and thickness of the cortical bone [39]. A positive impact of the thickness of the crestal and the buccal cortical bone on the primary stability of implants was also observed in clinical studies [40]. In vitro, a positive correlation could be observed between the RFA value and the bone implant contact (BIC) [41]. However, this could not be demonstrated in animal experiments. Therefore, conclusive association between RFA values and the primary BIC has not been proven [42]. In a histological study, a significant positive correlation was observed between the bone volume and RFA values [43]. However, in the present study, no significant correlations were observed between the histomorphometric results and bone density measurements and between bone density measurements and the primary stability of implants.

Prognostic value of the primary stability on the survival of implants does not seem conclusive. In contrast, there is a significant correlation between the primary and the secondary stability [18]. Immediate loading of implants requires high primary stability [44]. Therefore, radiological preoperative assessment of primary stability would be helpful in treatment planning. Further conclusive clinical studies regarding bone quality assessed by CBCT scans need to be carried out.

## Figures and Tables

**Table 1:** Distribution of the teeth of 87 patients according to their positions in the jaw.

Region.	ARP Group	Control Group	Total
Anterior teeth	17	17	34
Premolars	19	21	40
Molars	8	5	13

ARP: alveolar ridge preservation.

**Table 2:** Bone density after tooth extraction in the alveolar ridge preservation group and the control group. Mean values of the respective sectional planes were standardized with the average value of the local alveolar bone density measured in the palatal-apical direction (calculated as “Hounsfield-equivalents”).

Parameter	Group	Valid Datasets	Mini-Mum	25% Percentile	Median	75% Percentile	Maxi-Mum	*p*-Value
Upper third (1/3)	ARP	44	−13.03	−0.82	−0.40	0.14	7.59	0.55
	Control	43	−5.86	−0.61	−0.36	0.08	6.43	
Middle (1/2)	ARP	44	−10.49	−0.49	−0.22	0.25	3.43	0.78
	Control	43	−5.10	−0.36	−0.12	0.18	5.75	
Lower third (2/3)	ARP	44	−6.82	−0.40	0.14	0.33	1.27	0.70
	Control	43	−2.92	−0.13	0.15	0.37	5.85	

ARP: alveolar ridge preservation.

**Table 3:** Differences in bone density among different measurement locations in the alveolus according to the overall group, the ARP-group, and the control group including maxima, median, and minima. The difference of 0 was investigated using Wilcoxon signed-rank test and the group comparison was performed using Wilcoxon rank-sum test.

Parameter	Group	Valid Datasets	Mini-Mum	25% Percen-Tile	Median	75% Percen-Tile	Maxi-Mum	WSR *p*-Value	WRS *p*-Value
Difference 1/3 to 1/2	total	87	−2.55	−0.42	−0.13	0.00	4.16	<0.01	
	ARP	44	−2.55	−0.54	−0.21	0.01	4.16	<0.01	0.42
	control	43	−1.62	−0.35	−0.12	0.00	0.69	<0.01	
Difference 1/3 to 2/3	total	87	−6.21	−0.93	−0.34	−0.02	8.29	<0.01	
	ARP	44	−6.21	−0.94	−0.39	−0.02	8.29	<0.01	0.76
	control	43	−3.86	−0.86	−0.31	−0.06	1.65	<0.01	
Difference 1/2 to 2/3	total	87	−3.67	−0.45	−0.19	0,02	4.13	<0.01	
	ARP	44	−3.67	−0.56	−0.16	0.02	4.13	<0.01	0.68
	control	43	−2.24	−0.43	−0.28	−0.03	0.96	<0.01	

ARP: alveolar ridge preservation, WSR: Wilcoxon signed-rank test, WRS: Wilcoxon rank-sum test.

**Table 4:** Association between histomorphometric results and radiologically measured bone density as a function of measurement location.

Histomorphometric Parameters	Location of Bone Density Measurement	Correlation Coefficient	*p*-Value
Osteogenesis	1/3	−0.14	0.57
	½	−0.20	0.42
	2/3	−0.26	0.30
Remodelling	1/3	−0.18	0.46
	½	−0.26	0.30
	2/3	−0.42	0.08
Activity of osteoblasts	1/3	−0.27	0.27
	½	−0.27	0.29
	2/3	−0.27	0.28
Mineralization	1/3	0.16	0.53
	½	0.09	0.71
	2/3	−0.08	0.74
Bundle Bone	1/3	0.08	0.76
	½	0.03	0.90
	2/3	−0.11	0.66
Vascularization	1/3	−0.35	0.15
	½	−0.34	0.16
	2/3	−0.33	0.17
Inflammation yes/no	1/3	−0.44	0.06
	½	−0.40	0.09
	2/3	−0.37	0.11

**Table 5:** Distribution of the teeth of 55 patients according to their positions in the jaw.

Region	ARP Group	Control Group	Total
Anterior teeth	8	9	17
Premolars	15	14	29
Molars	6	3	9

ARP: alveolar ridge preservation.

**Table 6:** Distribution of the lengths and the diameters of the inserted implants.

Parameter	ARP Group			Control Group		
	Diameter					
	3.3 mm	3.8 mm	4.3 mm	3.3 mm	3.8 mm	4.3 mm
Length						
7 mm			1			
9 mm			5		1	5
11 mm		9	4		7	1
13 mm		9	1	1	10	1

ARP: alveolar ridge preservation.

**Table 7:** Primary stability of the implants in the ARP group and the control group.

Parameter	Group	Valid Datasets	Mean	SD	Minimum	Maximum	*p*-Value
ISQ buccal	ARP	29	63.00	8.75	35.00	73.00	0.69
	Control	26	64.12	7.88	41.00	74.00	
ISQ mesial	ARP	29	63.48	9.60	35.00	76.00	0.70
	Control	26	65.15	8.30	43.00	75.00	

ARP: alveolar ridge preservation, SD: standard deviation, ISQ: implant stability quotient.

**Table 8:** Association between primary stability and radiologically measured bone density as a function of the measurement location.

Parameter	Location of Bone Density Measurement	Spearman’s Rank Correlation Coefficient (ρ)	*p*-Value
ISQ buccal	1/3	−0.04	0.75
	2/3	0.10	0.48
	3/3	0.22	0.11
ISQ mesial	1/3	−0.03	0.81
	2/3	0.10	0.48
	3/3	0.21	0.12

ISQ: implant stability quotient.
